# 1059. *In vitro* Activity of Exebacase (CF-301) against *Staphylococcus aureus* Causing Bacteremia in the United States, Including Multidrug-resistant Subsets

**DOI:** 10.1093/ofid/ofab466.1253

**Published:** 2021-12-04

**Authors:** Rodrigo E Mendes, Jill Lindley, Nabina Gurung, Mariana Castanheira, Mariana Castanheira, Ray Schuch, Jane E Ambler

**Affiliations:** 1 JMI Laboratories, North Liberty, Iowa; 2 ContraFect, Yonkers, New York

## Abstract

**Background:**

Exebacase (CF-301) is a lysin (peptidoglycan hydrolase enzyme) with anti-staphylococcal activity. CF-301 is in Phase 3 of clinical development for the treatment of *Staphylococcus aureus* (SA) bacteremia (SAB), including right-sided infective endocarditis (IE), used in addition to standard-of-care antibiotics. CF-301 *in vitro* activity was determined against SA isolates reflecting the Phase 3 target patient SAB population, including IE.

**Methods:**

666 SA recovered from blood (3% from known IE) of patients hospitalized in 29 centers located in 9 Census regions were included as part of the SENTRY Antimicrobial Surveillance Program for 2020. Identification was confirmed by MALDI-TOF. Susceptibility to 12 comparators used reference broth microdilution (BMD), whereas CF-301 used a modified BMD method with cation-adjusted Mueller-Hinton broth (CAMHB) supplemented with 25% horse serum and 0.5 mM DL-dithiothreitol according to CLSI. MIC interpretation for comparator agents used CLSI criteria, including determination of multidrug-resistant (MDR) phenotype (non-susceptible to ≥3 classes of antibiotics).

**Results:**

Against all SA tested CF-301 had an MIC range of 0.06-1 mg/L, with MIC_50_, MIC_90_ and modal MIC values of 0.5 mg/L. CF-301 MIC results (MIC_50/90_, 0.5/0.5 mg/L) against methicillin-susceptible (MSSA) and -resistant (MRSA; 38.6% of all SA) SA were identical. Many comparators had activity against MSSA; among drugs indicated for treating SAB caused by MRSA, daptomycin and vancomycin were active (100% susceptible) against all isolates. A total of 62.3% of MRSA isolates were categorized as MDR, and CF-301 showed equal MIC_50_ and MIC_90_ results against MDR (MIC_50/90_, 0.5/0.5 mg/L) and non-MDR (MIC_50/90_, 0.5/0.5 mg/L) populations. Daptomycin and vancomycin were active (100% susceptible) against MDR MRSA isolates.

**Conclusion:**

CF-301 was uniformly active against contemporary SA isolates responsible for bloodstream infections in the US in 2020. CF-301 activity was consistent, regardless of resistance phenotype (MSSA, MRSA, including MDR isolates). Surveillance data presented here further support the clinical development of CF-301 as a promising option for treatment of SAB, including those caused by MDR MRSA isolates.

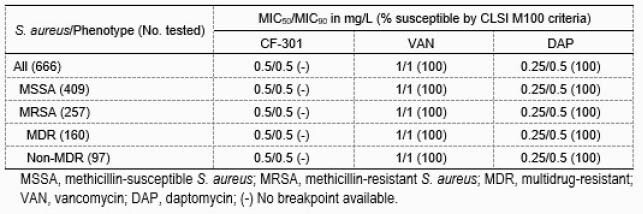

**Disclosures:**

**Rodrigo E. Mendes, PhD**, **AbbVie** (Research Grant or Support)**AbbVie (formerly Allergan**) (Research Grant or Support)**Cipla Therapeutics** (Research Grant or Support)**Cipla USA Inc.** (Research Grant or Support)**ContraFect Corporation** (Research Grant or Support)**GlaxoSmithKline, LLC** (Research Grant or Support)**Melinta Therapeutics, Inc.** (Research Grant or Support)**Melinta Therapeutics, LLC** (Research Grant or Support)**Nabriva Therapeutics** (Research Grant or Support)**Pfizer, Inc.** (Research Grant or Support)**Shionogi** (Research Grant or Support)**Spero Therapeutics** (Research Grant or Support) **Jill Lindley**, **Bravos Biosciences** (Research Grant or Support)**ContraFect Corporation** (Research Grant or Support)**Pfizer, Inc.** (Research Grant or Support)**Qpex Biopharma** (Research Grant or Support) **Nabina Gurung, n/a**, **ContraFect Corporation** (Research Grant or Support) **Mariana Castanheira, PhD**, **AbbVie (formerly Allergan**) (Research Grant or Support)**Bravos Biosciences** (Research Grant or Support)**Cidara Therapeutics, Inc.** (Research Grant or Support)**Cipla Therapeutics** (Research Grant or Support)**Cipla USA Inc.** (Research Grant or Support)**GlaxoSmithKline** (Research Grant or Support)**Melinta Therapeutics, Inc.** (Research Grant or Support)**Melinta Therapeutics, LLC** (Research Grant or Support)**Pfizer, Inc.** (Research Grant or Support)**Qpex Biopharma** (Research Grant or Support)**Shionogi** (Research Grant or Support)**Spero Therapeutics** (Research Grant or Support) **Mariana Castanheira, PhD**, Affinity Biosensors (Individual(s) Involved: Self): Research Grant or Support; Allergan (Individual(s) Involved: Self): Research Grant or Support; Amicrobe, Inc (Individual(s) Involved: Self): Research Grant or Support; Amplyx Pharma (Individual(s) Involved: Self): Research Grant or Support; Artugen Therapeutics USA, Inc. (Individual(s) Involved: Self): Research Grant or Support; Astellas (Individual(s) Involved: Self): Research Grant or Support; Basilea (Individual(s) Involved: Self): Research Grant or Support; Beth Israel Deaconess Medical Center (Individual(s) Involved: Self): Research Grant or Support; BIDMC (Individual(s) Involved: Self): Research Grant or Support; bioMerieux Inc. (Individual(s) Involved: Self): Research Grant or Support; BioVersys Ag (Individual(s) Involved: Self): Research Grant or Support; Bugworks (Individual(s) Involved: Self): Research Grant or Support; Cidara (Individual(s) Involved: Self): Research Grant or Support; Cipla (Individual(s) Involved: Self): Research Grant or Support; Contrafect (Individual(s) Involved: Self): Research Grant or Support; Cormedix (Individual(s) Involved: Self): Research Grant or Support; Crestone, Inc. (Individual(s) Involved: Self): Research Grant or Support; Curza (Individual(s) Involved: Self): Research Grant or Support; CXC7 (Individual(s) Involved: Self): Research Grant or Support; Entasis (Individual(s) Involved: Self): Research Grant or Support; Fedora Pharmaceutical (Individual(s) Involved: Self): Research Grant or Support; Fimbrion Therapeutics (Individual(s) Involved: Self): Research Grant or Support; Fox Chase (Individual(s) Involved: Self): Research Grant or Support; GlaxoSmithKline (Individual(s) Involved: Self): Research Grant or Support; Guardian Therapeutics (Individual(s) Involved: Self): Research Grant or Support; Hardy Diagnostics (Individual(s) Involved: Self): Research Grant or Support; IHMA (Individual(s) Involved: Self): Research Grant or Support; Janssen Research & Development (Individual(s) Involved: Self): Research Grant or Support; Johnson & Johnson (Individual(s) Involved: Self): Research Grant or Support; Kaleido Biosceinces (Individual(s) Involved: Self): Research Grant or Support; KBP Biosciences (Individual(s) Involved: Self): Research Grant or Support; Luminex (Individual(s) Involved: Self): Research Grant or Support; Matrivax (Individual(s) Involved: Self): Research Grant or Support; Mayo Clinic (Individual(s) Involved: Self): Research Grant or Support; Medpace (Individual(s) Involved: Self): Research Grant or Support; Meiji Seika Pharma Co., Ltd. (Individual(s) Involved: Self): Research Grant or Support; Melinta (Individual(s) Involved: Self): Research Grant or Support; Menarini (Individual(s) Involved: Self): Research Grant or Support; Merck (Individual(s) Involved: Self): Research Grant or Support; Meridian Bioscience Inc. (Individual(s) Involved: Self): Research Grant or Support; Micromyx (Individual(s) Involved: Self): Research Grant or Support; MicuRx (Individual(s) Involved: Self): Research Grant or Support; N8 Medical (Individual(s) Involved: Self): Research Grant or Support; Nabriva (Individual(s) Involved: Self): Research Grant or Support; National Institutes of Health (Individual(s) Involved: Self): Research Grant or Support; National University of Singapore (Individual(s) Involved: Self): Research Grant or Support; North Bristol NHS Trust (Individual(s) Involved: Self): Research Grant or Support; Novome Biotechnologies (Individual(s) Involved: Self): Research Grant or Support; Paratek (Individual(s) Involved: Self): Research Grant or Support; Pfizer (Individual(s) Involved: Self): Research Grant or Support; Prokaryotics Inc. (Individual(s) Involved: Self): Research Grant or Support; QPEX Biopharma (Individual(s) Involved: Self): Research Grant or Support; Rhode Island Hospital (Individual(s) Involved: Self): Research Grant or Support; RIHML (Individual(s) Involved: Self): Research Grant or Support; Roche (Individual(s) Involved: Self): Research Grant or Support; Roivant (Individual(s) Involved: Self): Research Grant or Support; Salvat (Individual(s) Involved: Self): Research Grant or Support; Scynexis (Individual(s) Involved: Self): Research Grant or Support; SeLux Diagnostics (Individual(s) Involved: Self): Research Grant or Support; Shionogi (Individual(s) Involved: Self): Research Grant or Support; Specific Diagnostics (Individual(s) Involved: Self): Research Grant or Support; Spero (Individual(s) Involved: Self): Research Grant or Support; SuperTrans Medical LT (Individual(s) Involved: Self): Research Grant or Support; T2 Biosystems (Individual(s) Involved: Self): Research Grant or Support; The University of Queensland (Individual(s) Involved: Self): Research Grant or Support; Thermo Fisher Scientific (Individual(s) Involved: Self): Research Grant or Support; Tufts Medical Center (Individual(s) Involved: Self): Research Grant or Support; Universite de Sherbrooke (Individual(s) Involved: Self): Research Grant or Support; University of Iowa (Individual(s) Involved: Self): Research Grant or Support; University of Iowa Hospitals and Clinics (Individual(s) Involved: Self): Research Grant or Support; University of Wisconsin (Individual(s) Involved: Self): Research Grant or Support; UNT System College of Pharmacy (Individual(s) Involved: Self): Research Grant or Support; URMC (Individual(s) Involved: Self): Research Grant or Support; UT Southwestern (Individual(s) Involved: Self): Research Grant or Support; VenatoRx (Individual(s) Involved: Self): Research Grant or Support; Viosera Therapeutics (Individual(s) Involved: Self): Research Grant or Support; Wayne State University (Individual(s) Involved: Self): Research Grant or Support **Ray Schuch, PhD**, **ContraFect Corporation** (Employee) **Jane E. Ambler, PhD**, **ContraFect Corporation** (Employee)

